# Beyond glycemic control: a holistic perspective on psychosocial support in outpatient diabetes management

**DOI:** 10.3389/fendo.2025.1708620

**Published:** 2025-11-27

**Authors:** Lin-na Hao, Xiao-wei Ma, Li-na Kang, Yu-ying Wang, Hong Shi

**Affiliations:** 1 Outpatient Department, Hongqi Hospital Affiliated to Mudanjiang Medical University, Mudanjiang, China; 2 Department of Intensive Care Unit, Hongqi Hospital Affiliated to Mudanjiang Medical University, Mudanjiang, China; 3 Blood Draw Room, Hongqi Hospital Affiliated to Mudanjiang Medical University, Mudanjiang, China; 4 Department of Endocrinology, Hongqi Hospital Affiliated to Mudanjiang Medical University, Mudanjiang, China; 5 Department of Rheumatology and Immunology, Hongqi Hospital Affiliated to Mudanjiang Medical University, Mudanjiang, China

**Keywords:** diabetes mellitus, diabetes management, psychosocial support, biopsychosocial model, outpatient care, integrated care

## Abstract

Conventional outpatient diabetes management, which focuses mainly on biomedical measures like glycemic control, may be inadequate for achieving sustainable long-term health outcomes, especially among patients with co-occurring psychosocial challenges. Although these methods are physiologically important, they often ignore key psychosocial factors that greatly affect self-management, treatment adherence, and clinical results. Based on the biopsychosocial model, this article proposes a comprehensive care framework that includes structured psychosocial support as a core part of diabetes management. Strong evidence shows that psychosocial factors—such as diabetes-related distress, mental health conditions, and social determinants—directly influence glycemic control, quality of life, and complication rates. The article also points out structural weaknesses in current healthcare systems that prevent integrated care. In response, a coordinated, multi-level strategy is introduced. This includes systematic psychosocial screening, communication methods supported by evidence, digital health technologies, and personalized stepped-care interventions. Finally, we recommend systemic reforms in clinical practice, payment policies, and medical education to support a shift toward person-centered, biopsychosocial diabetes care. These changes are necessary to address the complex nature of diabetes and improve both health outcomes and patient well-being.

## Highlights

This article critiques the prevailing biomedical paradigm in outpatient diabetes care and advocates for a comprehensive biopsychosocial approach. It demonstrates that exclusive focus on glycemic metrics fails to address critical psychosocial dimensions—including diabetes-specific distress, mental health comorbidities, and social determinants—that substantially influence self-management behaviors, treatment adherence, and long-term health outcomes. The analysis identifies multilevel barriers to integrated care at systemic, practitioner, and patient levels, while proposing an implementable framework featuring systematic psychosocial assessment, evidence-based communication strategies, stratified interventions, and digital health integration. The manuscript concludes by urging transformative reforms across clinical practice, payment structures, and medical education to advance person-centered diabetes care that effectively addresses both physiological and psychosocial aspects of health.

## 1 Introduction

The global diabetes epidemic continues to escalate, presenting one of the most significant public health challenges of the 21st century and creating substantial economic and social burdens for healthcare systems worldwide (1, 2). Conventional outpatient diabetes management remains largely biomedically oriented, emphasizing glycemic monitoring, pharmaceutical treatment, and basic lifestyle adjustments (3). While these components form the foundation of glycemic regulation, their predominant focus fails to address the broader human experience of chronic disease management (4).

This therapeutic approach invites a crucial clinical question: Does meeting HbA1c targets necessarily constitute successful diabetes management? (5) Accumulating evidence indicates that even patients with excellent glycemic control may still experience diminished quality of life, significant emotional distress, and unexpected complication risks (5, 6). These observations reveal a critical limitation in prevailing treatment paradigms—the systematic oversight of psychosocial factors (7). Although leading organizations such as the American Diabetes Association and the American Association of Clinical Endocrinology have begun to formally endorse the assessment of psychosocial factors, social determinants of health, and diabetes distress in their recent guidelines (8–10), the systematic adoption of these practices remains limited and inconsistent. The concept of “diabetes distress” has gained recognition to describe the distinctive emotional burdens and chronic anxieties associated with continuous self-management demands (11, 12). Such psychological factors substantially impact medication adherence, self-care behaviors, and long-term health outcomes.

In light of these evidence-based insights, this perspective contends that contemporary diabetes management must transcend conventional biochemical metrics to adopt an integrative, person-centered approach. The incorporation of systematic psychosocial support into standard care protocols represents not merely an enhancement but a necessity for comprehensive diabetes care. This reconceptualization positions the individual—not just the disease—at the center of therapeutic efforts. A multidimensional framework that synergistically combines biological, psychological, and social elements is fundamental to achieving sustainable health outcomes, reducing diabetes-related distress, and fulfilling the principles of whole-person care.

## 2 Need to move beyond glycemic control: critical role of psychosocial dimensions

### 2.1 The bidirectional relationship between diabetes and mental health

#### 2.1.1 Diabetes as a chronic psychological stressor

The persistent demands of diabetes self-management represent a significant and ongoing source of psychological stress. The continuous requirements for glycemic monitoring, strict medication adherence, dietary modifications, and the pervasive fear of potential complications collectively contribute to a substantial mental health burden (13–15). This burden frequently manifests as diabetes-specific distress, characterized by feelings of being overwhelmed, frustrated, and emotionally drained by the relentless nature of disease management (13, 14). When unaddressed, this condition may progress to more severe psychological comorbidities, including major depressive disorder and generalized anxiety disorder (14, 15).

#### 2.1.2 Psychological comorbidities and their physiological consequences

Psychological disorders exert considerable negative effects on diabetes progression through multiple pathways. Depression and anxiety can induce neurohormonal alterations, including dysregulation of the hypothalamic-pituitary-adrenal axis and increased sympathetic nervous system activity, which promote insulin resistance and hyperglycemia (16). Additionally, these conditions negatively influence health behaviors, reducing medication adherence, impairing dietary management, and decreasing physical activity levels (11, 17). This establishes a detrimental cycle wherein psychological distress worsens metabolic control, which in turn amplifies emotional suffering and increases vulnerability to diabetes complications.

### 2.2 The fundamental influence of social determinants

#### 2.2.1 The protective role of social support systems

Comprehensive social support networks—including family, peers, and community resources—serve essential functions in diabetes management (18, 19). These networks provide emotional support through empathy and encouragement, practical assistance with daily management tasks, and valuable information sharing (20). Evidence consistently demonstrates that strong social support correlates with improved treatment adherence, enhanced psychological well-being, and more favorable clinical outcomes, thereby serving as a crucial buffer against the challenges of chronic disease management (20, 21).

#### 2.2.2 Socioeconomic disparities in diabetes outcomes

Socioeconomic status remains a powerful determinant of diabetes management success. Income level, educational attainment, health literacy, and access to affordable healthcare services collectively determine an individual’s capacity to implement effective self-care strategies (22). Socioeconomic disadvantages often create substantial barriers to optimal diabetes care, including financial constraints, limited access to healthy food options, and inadequate health insurance coverage, frequently resulting in delayed diagnoses, suboptimal treatment, and disproportionately adverse health outcomes (23–25).

#### 2.2.3 Cultural influences on health beliefs and behaviors

Cultural background significantly shapes individuals’ perceptions of diabetes, influencing their understanding of disease etiology, treatment preferences, and health-seeking behaviors (26, 27). Culturally mediated health beliefs may affect patients’ acceptance of biomedical explanations and their willingness to engage with recommended treatment regimens (26, 28, 29). Recognizing and respecting these cultural perspectives is essential for developing culturally sensitive care approaches, facilitating collaborative treatment planning, and establishing effective patient-provider communication, ultimately leading to improved patient engagement and treatment adherence (27, 28, 30).

## 3 Identified gaps and challenges in contemporary outpatient diabetes care

### 3.1 System-level obstacles

Current healthcare delivery systems present fundamental structural barriers to integrating psychosocial support into diabetes management. Limited consultation durations in outpatient settings frequently preclude comprehensive assessment of non-medical issues (31). The pervasive fee-for-service payment model creates financial disincentives for providing time-intensive psychosocial services while preferentially reimbursing procedural interventions (32). Moreover, most healthcare environments lack properly constituted multidisciplinary teams capable of delivering integrated biopsychosocial care (33, 34). This systemic deficiency in care coordination mechanisms and resource allocation fundamentally constrains the implementation of holistic treatment approaches (35).

### 3.2 Healthcare practitioner-related challenges

Significant clinician-related barriers further impede the adoption of psychosocial care modalities. Many medical professionals receive insufficient training in mental health screening, patient-centered communication strategies, and appropriate referral protocols for psychological support (36, 37). This educational gap often coincides with the perception that addressing emotional and social concerns exceeds their professional responsibilities—a view reinforced by conventional biomedical training paradigms (36–38). Practical limitations, including unfamiliarity with available mental health resources and community support services, further complicate appropriate patient referrals (37, 38). The high prevalence of professional burnout among diabetes care providers additionally compromises their capacity for empathetic engagement and psychosocial assessment during clinical interactions (36, 37, 39).

### 3.3 Patient-centered barriers

Individuals with diabetes encounter substantial personal and socio-cultural obstacles to seeking and accepting psychosocial support. Pervasive stigma surrounding mental health conditions frequently inhibits disclosure of psychological distress, as patients may anticipate negative judgment or perceive such discussions as irrelevant to medical care (40, 41). Common misconceptions include the belief that emotional difficulties represent inevitable accompaniments to chronic disease rather than addressable therapeutic concerns (40, 42). Cultural norms that prioritize physical health over psychological well-being, along with varying health literacy levels, may further discourage help-seeking behaviors (40, 43). These multifaceted barriers often result in unexpressed needs and unaddressed comorbidities that substantially compromise self-management efficacy and treatment outcomes (43, 44).

## 4 Developing an integrated psychosocial support framework: bridging theory and practice

### 4.1 Core principles

A robust psychosocial support framework in diabetes management rests upon three established pillars: person-centered care, empowerment strategies, and multidisciplinary collaboration. Person-centered care prioritizes understanding and incorporating patients’ unique values, preferences, and life circumstances into collaboratively developed management strategies (45, 46). Empowerment strategies focus on developing patients’ self-efficacy, problem-solving abilities, and confidence through structured education and skill development (47, 48). Multidisciplinary collaboration necessitates the coordinated involvement of endocrinologists, diabetes educators, mental health professionals, and social workers to address the biological, psychological, and social dimensions of diabetes care (45, 48).

### 4.2 Implementation strategies and methodologies

#### 4.2.1 Systematic psychosocial assessment

The integration of validated screening instruments into routine clinical practice enables early detection of psychosocial concerns. Standardized measures including the Problem Areas in Diabetes Scale (PAID-5) for diabetes-specific distress, the Patient Health Questionnaire (PHQ-9) for depressive symptoms, and multidimensional diabetes distress scales provide reliable mechanisms for identifying patients requiring additional support (49, 50). These assessments should be administered at regular intervals to monitor changes in psychosocial status.

#### 4.2.2 Evidence-based communication approaches

Effective clinician-patient interactions employ established therapeutic techniques including motivational interviewing to facilitate behavior change, shared decision-making to promote treatment engagement, and cognitive-behavioral strategies to address diabetes-related negative thinking patterns (51–53). These communication methods have demonstrated effectiveness in improving patient adherence and self-management capabilities.

#### 4.2.3 Stratified care delivery model

A stepped-care approach ensures efficient resource allocation matched to individual needs. Universal foundational support (Tier 1) encompasses diabetes education, peer support opportunities, and basic community resource navigation for all patients (54, 55). Targeted interventions (Tier 2) provide structured problem-solving therapy, stress reduction techniques, and coping skills training for individuals experiencing moderate distress (54, 55). Specialized mental health referral (Tier 3) offers access to psychiatric care and psychological therapies for patients with significant psychological comorbidities (54, 56).

#### 4.2.4 Digital health integration

Technology-enabled solutions significantly expand the reach and continuity of psychosocial support (57, 58). Mobile health applications, telehealth platforms, and virtual support communities provide accessible avenues for education, monitoring, and social connection (59, 60). These digital tools complement traditional care delivery by offering scalable support mechanisms that transcend geographical and temporal constraints.

### 4.3 Implementation considerations

Successful framework implementation requires organizational commitment, staff competency development, and sustainable funding mechanisms (61–63). Healthcare systems must establish clear protocols for screening, referral pathways, and interdisciplinary collaboration (61–63). Long-term viability depends on demonstrating improved clinical outcomes, enhanced patient experiences, and economic efficiency through rigorous evaluation and continuous quality improvement processes (64).

## 5 Advancing a paradigm shift: strategic recommendations and future directions

### 5.1 Clinical practice transformation

Integrating psychosocial care into standard diabetes management requires fundamental changes in clinical protocols. Evidence-based psychosocial assessments should be systematically embedded into routine clinical pathways to ensure consistent evaluation of psychological well-being and social determinants of health (62). Healthcare providers need dedicated training in mental health screening, patient-centered communication strategies, and basic supportive counseling techniques (62, 65). Additionally, developing formally structured collaboration mechanisms between medical institutions, community organizations, and family support systems is essential for creating continuous, integrated care networks that address patients’ comprehensive needs (65, 66).

### 5.2 Health policy and reimbursement reform

Strategic payment reform serves as a crucial catalyst for sustainable psychosocial care integration. Transitioning toward value-based payment models that incorporate patient-reported outcomes and quality-of-life metrics into performance assessment and reimbursement frameworks will create aligned incentives for whole-person care (64, 67, 68). Policy makers must simultaneously mandate adequate insurance coverage for validated psychosocial services—including mental health support, diabetes self-management education, and community-based programs—to ensure financial accessibility and eliminate coverage disparities (64, 69).

### 5.3 Educational advancement and research priorities

Medical education continues to require evolution to fully reflect contemporary healthcare needs. While many medical schools and residency programs have made commendable progress in incorporating topics such as social determinants of health, biopsychosocial models, and patient-centered communication skills into their curricula, the depth, consistency, and application of this training vary widely (70–72). Therefore, further modernization and standardization are imperative to ensure all future clinicians are proficient in providing holistic, person-centered diabetes care. The research agenda must prioritize large-scale effectiveness trials examining the long-term impact of psychosocial interventions on clinical outcomes, cost-effectiveness, and implementation sustainability (73). Further development and validation of culturally responsive assessment tools and intervention strategies for diverse populations represents an additional critical research imperative (74).

This multidimensional transformation requires coordinated commitment across clinical, educational, policy, and research domains. Through systematic implementation of these strategic recommendations, healthcare systems can progress toward more comprehensive, equitable, and effective diabetes care that addresses both biological and psychosocial dimensions of health.

## Data Availability

The original contributions presented in the study are included in the article/Supplementary Material. Further inquiries can be directed to the corresponding author.
